# Two-rope method for dissecting esophagus in McKeown MIE

**DOI:** 10.3389/fsurg.2022.1031142

**Published:** 2023-01-06

**Authors:** Qian Wang, Huibing Liu, Luchang Zhang, Defeng Jin, Zhaoqing Cui, Rongqiang Cai, Junjun Huang, Yutao Wei

**Affiliations:** ^1^ School of Clinical Medicine, Jining Medical University, Jining, China; ^2^ Department of Thoracic and Cardiovascular Surgery, Nantong No. 1 People's Hospital, Nantong, China; ^3^ Department of Thoracic Surgery, Jining No. 1 People's Hospital, Jining, China; ^4^ Institute of Thoracic Surgery, Jining Medical Research Academy, Jining, China

**Keywords:** minimally invasive esophagectomy, esophagectomy, esophagus suspension method, thoracoscope, esophageal carcinoma

## Abstract

**Objective:**

Minimally invasive McKeown esophagectomy (McKeown MIE) is performed at many hospitals in esophageal cancer(EC) treatment. However, secure and quick methods for dissecting the esophagus and dissecting lymph nodes in this surgery are lacking. This study introduces a simple, secure and feasible esophagus dissecting technique named two-rope method. Two mobile traction ropes are placed around the esophagus and we tow these ropes to free the esophagus, dissect the lymph nodes, and decrease the operative trauma.

**Materials and Methods:**

Retrospective analysis was performed on 112 patients who underwent McKeown MIE in our center from January 2019 to September 2021. They were assigned into two groups based on the method of dissecting the esophagus: Group A (two-rope method, 45 cases) and Group B (regular method, 67 cases). Operation time, thoracic operation time, the number of dissected thoracic lymph nodes, and postoperative complications were compared between the two groups after propensity score matching.

**Results:**

Using 1:1 nearest neighbor matching, we successfully matched 41 pairs of patients. Operation time, thoracic operation time, and the duration (ac to as) was significantly shorter and the size of the abdominal incision was significantly smaller in the Group A than Group B (*p *< 0.05). There was no statistically significant difference in the number of dissected thoracic lymph nodes, pulmonary infection, anastomotic leak, recurrent laryngeal (RLN) injury, and chylothorax between the two groups (*p *> 0.05).

**Conclusions:**

Two-rope method to free the esophagus and dissect thoracic lymph nodes in McKeown MIE has significant advantages compared with the regular method. The technique is, therefore suitable for widespread adoption by surgeons.

## Introduction

Esophageal cancer (EC) is the sixth most common cause of cancer-related death worldwide (1). The incidence and mortality of esophageal cancer in China are higher than the global average (2). Esophagectomy, including complete primary tumor removal and radical lymphadenectomy with or without cervical lymphadenectomy, has been accepted as a radical esophagectomy that remains a standard treatment choice for esophageal squamous cell carcinoma (ESCC) (3). Earlier studies have reported low incidences rate of pulmonary infection and mortality, and better long-term survival in minimally invasive esophagectomy (MIE), compared with open esophagectomy (OE) (4–6). Since most esophageal cancers are squamous cell carcinoma (SCC) and located in the middle of the esophagus in China, minimally invasive McKeown esophagectomy (McKeown MIE) is preferred in our center (7).

Location and size of esophageal tumors are variable and meanwhile, freeing of esophagus as well as lymphadenectomy along the esophagus and recurrent laryngeals (RLNs), which have a high incidence of lymphatic metastasis in these regions, are fundamental steps of McKeown MIE (8, 9). Thus, surgeons should be able to expertly free the entire thoracic esophagus, and perform the dissection of lymph nodes along esophagus, left and right RLNs lymph nodes, and subtrochanteric lymph nodes in the thoracic procedure of the operation (10). However, McKeown MIE keeps a challenge. First, since freeing the esophagus and dissecting lymph nodes around the esophagus under the thoracoscope were difficult, surgeons often need to spend much time and energy to complete the standard surgical steps, which means a long anesthesia time and an enormous cardiorespiratory burden on the patient. Moreover, the anatomy of the aorta, thoracic duct, and trachea in the region of the left RLN is complex and variable, and it isn't easy to dissecting in the narrow space, which was indispensable in McKeown MIE. The exact dissection under the thoracoscope frequently takes more time than OE (5, 6), and may irritate RLNs.

In the past, some experts proposed a method to suspend and free esophagus (11). With the application of the suspension line, the upper esophagus could be suspended. And the surgeon could easily reveal the surgical field of the esophagus-trachea groove and the aortic arch and then free the upper thoracic esophagus, inferior lymph nodes of the aortic arch, the lymph nodes, and soft tissues surrounding the left RLN. This method is particularly advantageous for isolating the left RLN lymph nodes and upper esophagus.

Inspired by it, we strived to improve the esophageal suspension method during McKeown MIE. While performing the operating, two ropes were placed around the esophagus to suspend and free the entire thoracic esophagus and thoracic lymph nodes. The upper rope was used to release the upper thoracic esophagus, dissect the lymph nodes around the left RLN, and accurately locate the cervical esophagus. The lower rope was used to free the lower thoracic esophagus, accurately tow the free-esophagus from the thoracic cavity into the abdominal cavity through the esophageal hiatus and then quickly tow to the abdominal surface together with the free-stomach.

The purpose of this study was to propose a two-rope method for freeing the esophagus and dissecting thoracic lymph nodes in McKeown MIE. And then evaluate the feasibility of this method.

## Materials and methods

### Patients

We retrospectively analyzed the clinical data of 112 patients who underwent McKeown MIE from January 2019 to September 2021. Forty-five cases underwent McKeown MIE with the help of the two-rope method (Group A), while 67 cases were operated with the help of the regular method (Group B). All patients were diagnosed with ESCC by a gastroscopy biopsy before the operation and the clinical stage of the patients was determined by comprehensive physical and imaging examination, including contrast-enhanced CT of chest and upper abdomen and PET-CT. All operations were performed by an experienced thoracic surgeon. Data of intraoperative, demographics, and postoperative complications were analyzed retrospectively.

The inclusion criteria were: tumor stage I–III [International Union Against Cancer (UICC) Version 3, 2020]; the cardiopulmonary function was sufficient to allow for single-lung ventilation during the operation; complete clinical data.

The exclusion criteria for the patients were as follows: chemotherapy, radiotherapy and neoadjuvant therapy before surgery; upper esophageal tumor; inability to tolerate surgery; concomitant multiple operations; not willing to provide informed consent; anamnesis of thoracic diseases; cancer other than esophageal cancer; tuberculosis; silicosis.

This study was approved by the Clinical Ethics Committee of Jining No.1 hospital, Shandong province, China. All patients provided written informed consent.

### Data collection

Demographic and intraoperative data were collected retrospectively. Demographic data constituted age, sex, body mass index (BMI), tumor node metastasis (TNM) stage, tumor location (upper middle, or lower third), pathologic stage, and histology. Intraoperative data constituted operation time, thoracic operation time, the duration of the free-esophagus and free-stomach from the abdominal cavity to surface [Duration (ac to as)], the number of dissected lymph nodes in the thoracic cavity, and the length of the abdominal center incision. Operation time was defined as the time (min) from the first incision to final closure. The thoracic operation time was defined as the start of thoracic incision to the closure of the thoracic incision. The duration (ac to as) was defined as the time (s) required to pull out the severed free-esophagus and free-stomach completely to the abdomen surface after the abdominal center incision was made.

Postoperative complications included chylothorax, anastomotic leakage, pulmonary infection, and recurrent laryngeal nerve injury. We diagnose these complications by expert consensus (12).

### Surgical technique

**Figure 1:**
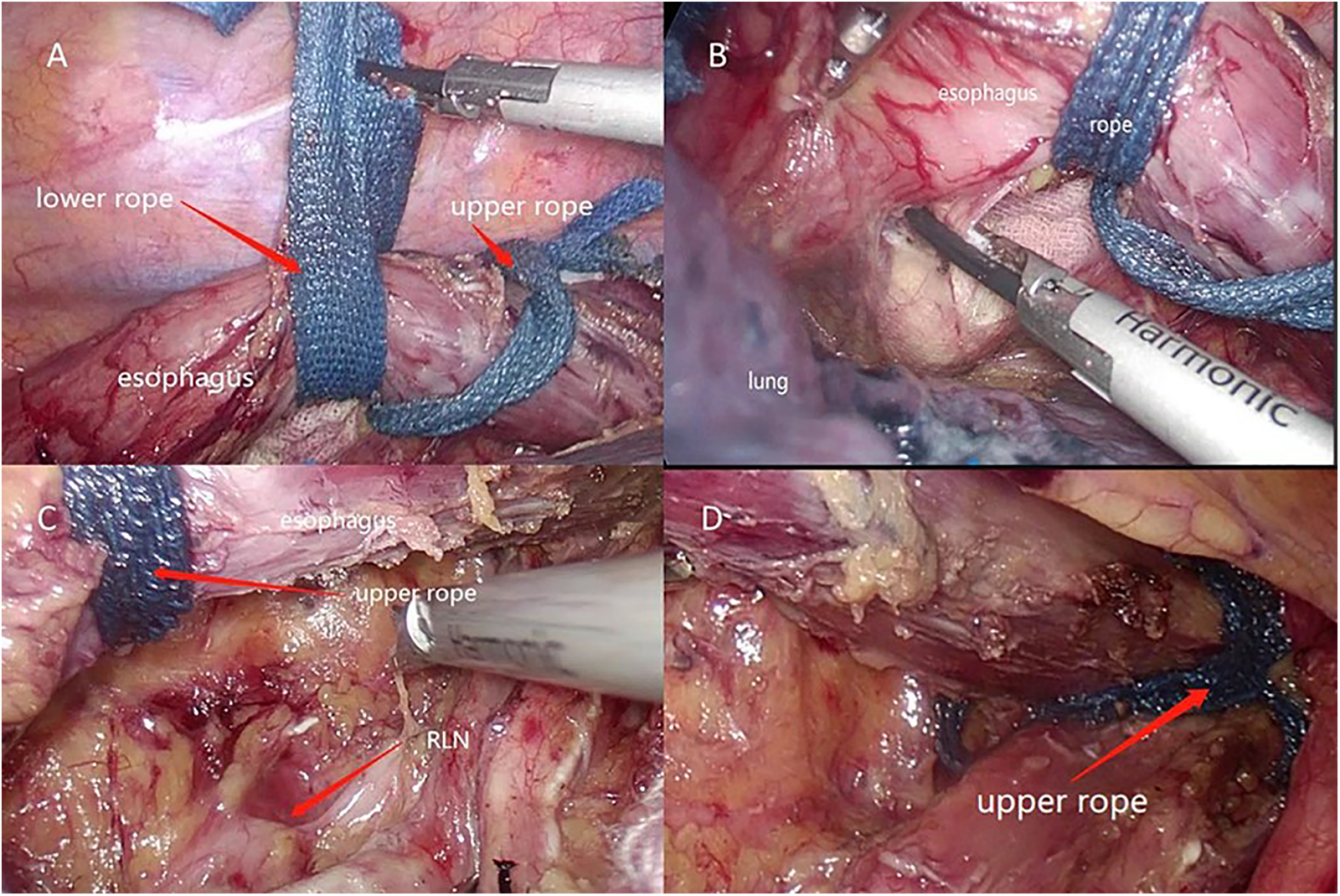
(**A**) Two ropes to free esophagus. (**B**) Suspend the esophagus to reveal the area of posterior esophagus and left pleura. (**C**) Tow the upper rope to enlarge the operation space. (**D**) Upper rope was placed in pleural roof.

Thoracic operation: After successful general anesthesia and double-lumen endotracheal intubation into the left lung for single lung ventilation. The patient was placed in the left lateral decubitus position, and operation holes were made on the 3 or 4th intercostal space of the anterior axillary line, 5th intercostal space of the posterior axillary line, and 8th intercostal space of the anterior axillary line. The thoracoscope was inserted into the 6th intercostal space of the mid-axillary line. The lungs were pushed forward, dissected the lymph nodes of the right RLN and the azygos arch was freed and cut. The posterior mediastinal pleura was opened with an electric hook. The esophagus was explored to determine the location of the tumor. The esophagus was freed 1–2 cm medially or above and below the esophageal tumor. And two mobile traction ropes were placed around the esophagus in these locations (Figure 1A). The esophagus was kept under traction ropes to provide more operating space in the posterior mediastinum. The surgeon towed the two mobile ropes to suspend the esophagus and dissect the adhesions of the esophagus and trachea, bronchus, hilum, and pericardium and remove mediastinal, para-esophageal, paratracheal, subcarinal, and lower pulmonary ligament lymph nodes. Meanwhile, towing ropes to control the esophagus helped to dissociate the tissue between the posterior esophagus and left pleura (Figure 1C). Finally, the surgeon hauled the upper rope to suspend and free the upper esophagus making it easier to hollow out the lymph nodes and connective tissue around the left RLN (Figure 1B). As the operation went on, the upper rope was pulled from the tumor or midpoint area to the pleural roof (Figure 1D) and the lower rope was removed to the esophageal hiatus for subsequent use. Thicker blood vessels or lymphatic vessels were cut with an ultrasonic knife. After ascertaining that there is no damage to the thoracic duct and RLNs, the chest was closed with an in-dwelling drain.

**Figure 2:**
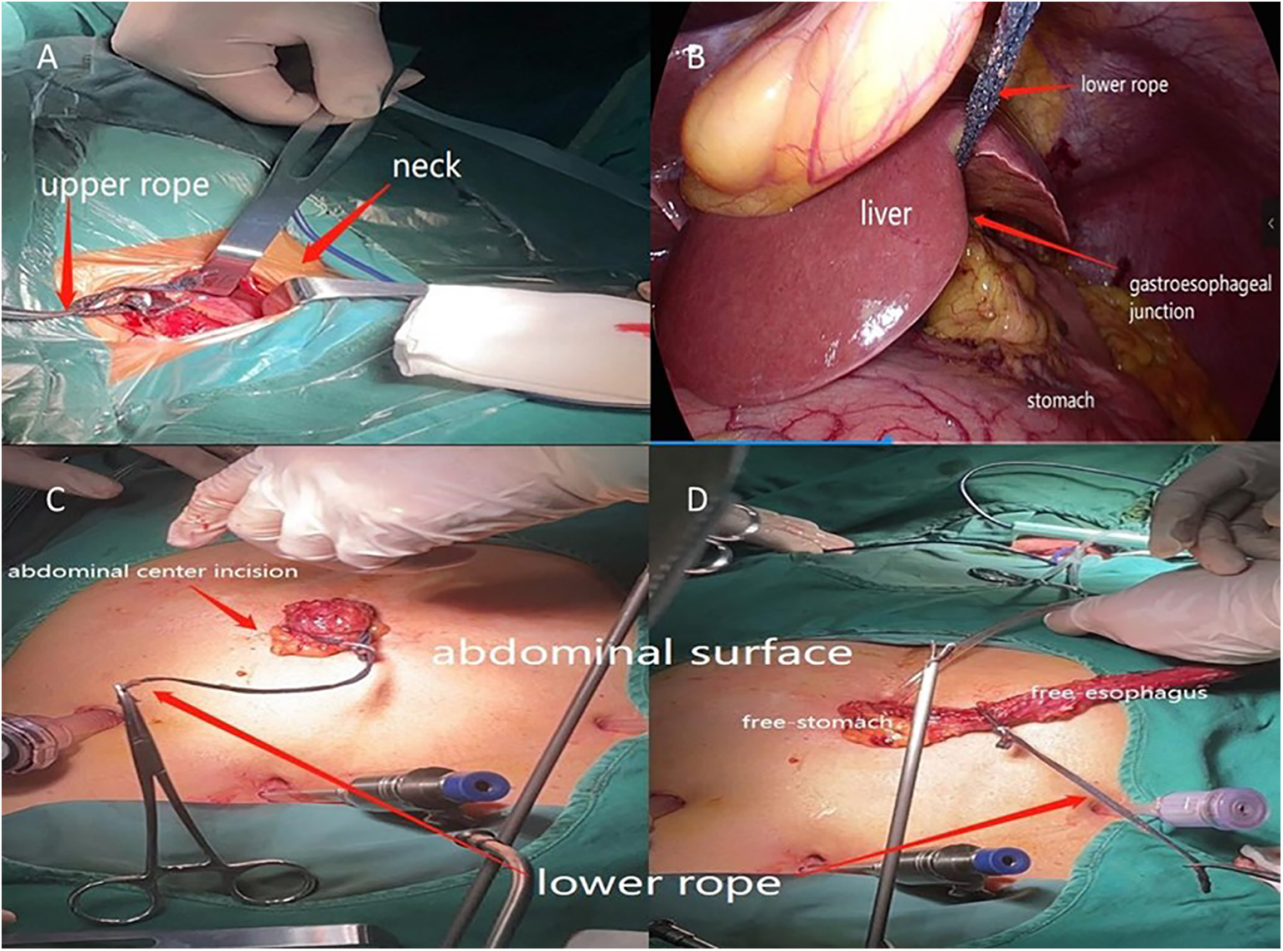
(**A**) Upper rope was used to locate and dissect the cervical esophagus. (**B**) Free-esophagus was towed by the lower rope to abdominal cavity. (**C**, **D**) Lower rope was used to tow the free-stomach and free-esophageal out of abdominal cavity.

Neck operation: The patient was placed in a supine position. An oblique incision (4 cm) was made on the cephalic side at the medial edge of the left sternocleidomastoid muscle. The cervical esophagus was located and freed by the blue upper rope in the neck operation (Figure 2B).

Abdominal operation: The patient was placed in a supine position. Artificial pneumoperitoneum was established. Dissect the stomach and the lymph nodes around the stomach. Once the cervical esophagus was transected, the lower rope was pulled from the thoracic cavity to the abdominal cavity through the esophageal hiatus together with the free-esophagus (Figure 2A). Once the abdominal center incision was made, the lower rope was towed together with the free-esophagus and free-stomach directly from the abdominal cavity to the surface (Figure 2C,D). And then the tubular stomach was created. The cutting stump was wrapped with a continuous suture. The tubular stomach was then drawn to the neck incision through the esophageal bed and a circular stapler was used to perform end-to-side anastomosis.

After all steps were completed, the abdominal incisions were sutured and the size of the incision on the center of abdomen was recorded.

### Statistical analysis

To address potential bias in the patients’ characteristics between the two groups, we used propensity score matching (PSM). Variables such as age, sex, BMI, comorbidities, tumor location and TNM stage were covariates. We created propensity score matching pairs with no replacement (1:1 matching) and set the caliper definition at 0.05. *x*^2^ test and Fisher's exact test were used for categorical data. Student's *t*-test was used for groups of data that were normally distributed, and the Mann–Whitney *U* test was used for non-normally distributed data. All statistical analyses were performed using SPSS 26 software (IBM Corp Armonk, NY). Values of *p* < 0.05 were considered statistically significant.

## Results

### Demographics

Forty-five patients underwent McKeown MIE with the help of the two-ropes method defined as Group A and 67 with the use of the regular method defined as Group B. Using 1:1 nearest neighbor matching, we successfully matched 41 pairs of patients. There was no significant difference in the demographics and clinical background between the two groups (*p *> 0.05) (Table 1).

**Table 1:** Characteristics of patients in two groups.

Characteristic	Before PSM		*p*	After PSM		*p*
	Group B, *n* = 67	Group A, *n* = 45		Group B, *n* = 41	Group A, *n* = 41	
Age, years	65.57 ± 7.43	64.46 ± 7.27	0.332	64.66 ± 7.45	65.12 ± 6.77	0.769
Sex, male/female	50/17	30/15	0.361	32/9	28/13	0.319
BMI	23.12 ± 3.22	23.45 ± 6.26	0.597	23.34 ± 3.26	23.61 ± 3.36	0.712
**Comorbidities**						
CVD	5	5	0.519	2	3	1
PD	4	2	1	3	2	1
T2DM	6	4	1	5	4	1
Tumor location			0.528			0.756
Upper segment	4	3		1	3	
Middle segment	37	20		21	20	
Lower segment	26	22		19	18	
Pathologic stage			0.626			0.628
0	2	0		2	0	
I	13	7		6	7	
II	26	16		16	14	
III	26	22		17	20	

CVD, cardiovascular disease; PD, pulmonary disease; T2DM: type 2 diabetes mellitus; BMI, body mass index; Group B, regular method to free esophagus; Group A, two-rope method to free esophagus.

### Intraoperative data

The intraoperative data for the two groups are presented in Table 2. All operations were R0 resection and no patient in the two groups were required to undergo OE. The total operation time of Group A was significantly lower than that of Group B (237.66 ± 30.34 min vs. 270.68 ± 43.10 min; *p* < 0.01). The thoracic operation time of Group A was also significantly lower than that of Group B (70.93 ± 8.88 min vs. 87.98 ± 14.28 min; *p* < 0.01). Similarly, the duration (ac to as) of Group A was significantly lower than that of the Group B (15.24 ± 3.81 s vs. 166.1 ± 28.19 s; *p *< 0.01). The length of the abdominal center incision was significantly smaller in Group A than that in Group B (4.01 ± 0.54 cm vs. 6.26 ± 1.09 cm; *p *< 0.01). There was no significant difference between the two group in the number of thoracic lymph nodes dissected and perioperative bleeding (*p *> 0.05).

**Table 2:** Intraoperative data in two-ropes group Bnd regular group.

Variable	Group B, *n* = 41	Group A, *n* = 41	*p*
the length of abdominal center incision, cm	6.26 ± 1.09	4.01 ± 0.54	0
Total operation time, min	270.68 ± 43.10	237.66 ± 30.34	0
Thoracoscopy time, min	87.98 ± 14.28	70.93 ± 8.88	0
Duration (ac to as), second	166.1 ± 28.19	15.24 ± 3.81	0
No. of thoracic lymph nodes removed	14.83 ± 3.89	14.44 ± 4.01	0.656
To open	0	0	1
R0	0	0	1

Duration (ac to as) was defined as the time (s) required to pull out the severed free-esophagus and free-stomach completely to the abdomen surface after the abdominal incision was made; Group B, regular method to free esophagus; Group A, two-rope method to free esophagus.

### Postoperative complications

Table 3 details the postoperative complications for the two groups. There was no statistically significant difference in anastomotic leakage (4 vs. 6. *p* > 0.05), pulmonary complications (5 vs. 9; *p* > 0.05), recurrent laryngeal nerve injury (1 vs. 1; *p* > 0.05) and chylothorax (1 vs. 1; *p* > 0.05) between two groups.

**Table 3:** Postoperative complications in two-ropes group Bnd regular group.

Variable	Group B, *n* = 41	Group A, *n* = 41	*p*
Pulmonary infection	9	5	0.24
Anastomotic leak	6	4	0.5
Recurrent laryngeal injury	1	1	1
Chylothorax	1	1	1

Group B, regular method to free esophagus; Group A, two-rope method to free esophagus.

## Discussion

McKeown MIE is an essential surgical procedure in EC treatment. As a safe and wide used surgical method, it enables the complete removal of the entire thoracic esophagus and radical lymph node dissection (5, 13). Compared with OE, MIE has been shown to shorten the risk of morbidity and mortality and be good in postoperative complications, lymph node dissection, blood loss and hospital stay (14, 15). However, there are still some problems troubling us. First, since freeing the esophagus and dissecting lymph nodes around the esophagus in thoracoscope, which were indispensable in McKeown MIE, were difficult, surgeons often need to spend more time and energy to complete the standard surgical steps. Moreover, the anatomy of the aorta, thoracic duct, and trachea in the region of the left RLN is variable. It is difficult to dissect in a narrow space (16). Finally, compared with the prone position, lung tissue is frequent to obscure the operative field in the left lateral decubitus position that we always choose. To solve the difficulty, some scholars have reported their valuable experience of freeing the esophagus and dissecting lymph nodes in McKeown MIE. Zheng et al. (11). described an esophageal suspension method. The surgeons could use one silk that was punctured out in the fifth intercostal space of the scapular inner edge to suspend the esophagus and then dissect the left RLN lymph nodes and the thoracic esophagus easily. This method not only reduces the probability of injury to the left RLN, but also increases the number of left RLN lymph nodes removed, which is corrected closely with the patient's health, pathological staging and prognosis (17). Zhang (18) et al. proposed the application of esophageal wire traction in McKeown MIE to dissect lymph nodes. Because of the use of a wire to generate traction, the esophagus was suspended. The wire could be towed to keep the esophagus under traction and to move the right lung forward, thereby increasing the working space in the posterior mediastinum and improving the stability of the video-assisted-thoracoscopy.

The two-rope method to suspend the esophagus proposed herewith is an improvement on the esophageal suspension method described by Zheng (11). First, application of the upper rope could assist in freeing and suspending the upper esophagus to dissect the lymph nodes and connective tissue around the left RLN during the thoracic operation (Figure 1B). Also, the upper rope could locate and free the cervical esophagus during the neck operation (Figure 2B). Finally, the lower rope could be used to free the lower esophagus and tow the free-esophagus and free-stomach from the thoracic cavity *via* abdominal cavity to surface through the abdominal center small incision (Figure 2C,D).

Compared with the Group B, the Group A had a significant advantage in operation time, including thoracic operation time, total operation time and the duration (ac to as). Why does Group A take less time? First, we think the two-rope method was significantly faster in freeing the thoracic esophagus and dissecting left RLN lymph nodes compared with the regular method. The surgeon could fully expose the esophageal bed by pulling the two ropes severally, making the anatomy of the thoracic esophagus clearer, speeding up the detachment of the esophagus and lymph nodes, and avoiding damage to the blood vessels and nerves. Pulling the upper ropes could expose the area of esophagus-trachea groove by suspending the esophagus to the right space, which could reduce the difficulty of dissection and increase the thoroughness of left RLN lymph nodes dissection, therefore the thoracic operation time was shorter in Group A than Group B. Then, since the lower rope was a long sterile rope, the surgeon could quickly and accurately drag the long rope out of the internal cavity (from thoracic cavity *via* abdominal cavity to surface) (Figure 2C,D) through abdominal center incision together with the free-stomach and free-esophagus after the abdominal incision was made. This method saves the process of probing the free-stomach by hand or oval forceps. Therefore, the duration (ac to as) was very short. Finally, by avoiding hand exploration, the surgeon could drag out the free-stomach with a smaller size abdominal center incision, requiring a shorter suture time. Thus, the total operation time was shorter than Group B. In a word, with this method, the surgeon could expose the operation field, speed up the detachment of the esophagus and lymph nodes, avoid directly touching the esophagus and stomach wall and tow the free-esophagus and free-stomach out of the abdominal cavity easily.

In regular McKeown MIE, it is primary for the assistant to repeatedly clamp the esophagus to help exposing the operation field. This may not only damage the esophageal muscular layer, increase intraoperative bleeding, and obstruct the visual field, but may also increase the surgeon's fatigue, increase the difficulty of the dissection, and waste time. And the surgeon always clamps the free-stomach and free-esophagus out of abdominal cavity through the abdominal center small incision by oval forceps or by hand directly for creating the tubular stomach outside during surgery. This is time-consuming, and increases the chances of accidental injury to the surrounding tissue and the stomach wall, and may cause complications such as intrathoracic hemorrhage, intraabdominal hemorrhage and tumor spread.

In esophagectomy, the number of lymph nodes dissected is closely related to prognosis and an essential index for evaluation of the quality of thoracoscopic esophagectomy (19, 20). We found that the accuracy of dissection and the number of thoracic lymph dissected by the two-ropes method was comparable with the regular method. It showed both methods were safe and reliable.

Pulmonary infection, anastomotic leakage, RLNs injury, and chylothorax are common complications after esophagectomy (21).Once happen, the patients can be challenging to solve and can ultimately be life-threatening in some situations. There was no statistically significant difference in these postoperative complications between the two groups. It indicated that there was no difference in the prognosis and postoperative recovery between the two groups. This further illustrated the effectiveness, safety, and feasibility of the two-rope method for freeing the esophagus in McKeown MIE.

## Conclusion

Two-rope method to free the esophagus and dissect lymph nodes in McKeown MIE has significant advantages compared with the regular method. The technique is therefore suitable for wide-spread adoption by the surgeons.

## Data Availability

The original contributions presented in the study are included in the article/Supplementary Material, further inquiries can be directed to the corresponding author/s.
